# Redox Control of Vascular Biology

**DOI:** 10.1155/2019/3764252

**Published:** 2019-07-22

**Authors:** J. Banerjee, J. Ghose, M. Sinha, S. Sen

**Affiliations:** ^1^George Washington University, Washington, DC, USA; ^2^The Ohio State University, Columbus, OH, USA; ^3^Indiana University, Indianapolis, IN, USA

Oxidative stress and nitrosative stress are defined as a disruption of redox signaling and control. Homeostatic concentrations of reactive oxygen species (ROS) and reactive nitrogen species (RNS) play a crucial role as secondary messengers in many intracellular signaling pathways; however, excess ROS can lead to cell and tissue injuries [1, 2]. The major sources of vascular ROS are NAD(P)H oxidase, mitochondrial-derived superoxide, and uncoupled nitric oxide synthase [3]. Increasing studies have shown a close relationship between angiogenesis and oxidative stress in both physiological and pathological conditions, and oxidative damage plays a major role in vascular dysfunction, leading to endothelial dysfunction and inflammation.

As the field of redox biology employs new techniques to quantitatively measure the amount of ROS/RNS generated, innovative strategies are being developed to therapeutically target redox imbalance in vascular disease. This issue consists of 7 papers highlighting different therapeutic approaches targeting redox damage in cardiomyopathy, hypertension, skin disease, and diabetic retinopathy.

L. Chang et al. studied the *role of ZYZ-803 molecule in acute myocardial infarction* (AMI). Their work showed that ZYZ-803 protects heart tissues against acute myocardial ischemia via the targeting of ERS-related necroptosis by downregulating the RIP3-CaMKII pathway. The molecule further regulates H_2_S and NO homeostasis via releasing both H_2_S and NO. This opens up avenues to test the molecule as a candidate in AMI therapeutics.

In the review by X. Yang et al., the authors discuss about *therapeutic application of traditional Chinese medicines for treating coronary heart diseases* (CHD). Increased oxidative stress, disturbed lipid metabolism, and increased inflammation are critical factors in the occurrence and development of atherosclerosis and subsequent CHD. The authors emphasize the unique advantage of traditional herbal medicine as they do not pose therapeutic side effects. Some of the herbs with medicinal properties as discussed by the authors include Ginseng, Astragalus, Rehmannia, Ophiopogon root, Rhodiola rosea, Codonopsis pilosula, Atractylodes macrocephala, Astragalus, and Fructus crataegi.

In the review by D. Xian et al., the authors provide an overview of the current knowledge of the link between *oxidative stress* (*OS*) *and angiogenesis and their roles in certain skin diseases*. The authors view that there are two main mechanisms implicated in the area bridging angiogenesis and OS. One is a VEGF-dependent signaling pathway, HIF/VEGF signaling, while another is a VEGF-independent signaling pathway (CEP/TLR2/MyD88 axis and ROS/ATM/p38*α* pathway). They opined that both OS and angiogenesis participate in the development of certain skin diseases like psoriasis and atopic dermatitis. A large spectrum of proangiogenic factors mediate in psoriasis, including VEGF, HIF-1*α*, TNF, angiopoietins, IL-8, IL-17, and TGF-*α*. VEGF could enhance the migration of leukocytes into psoriatic skin and increase oxygen consumption, further activating HIF-1*α* and perpetuating the angiogenic/inflammatory cycle of psoriasis. Thus, ROS-VEGF signaling may be a potential target for the treatment of psoriasis.

Diabetic retinopathy (DR) is a leading cause of visual impairment and morbidity around the world. Endothelial dysfunction in the retinal blood barrier accompanying the hyperglycemic state is considered to be the major insult for the onset and progression of DR [4]. In the review by N. Mahajan et al., the authors discuss *how impaired biochemical redox pathways contribute to diabetic retinopathy* highlighting the role of increased influx in polyol, accumulation of advanced end glycation products (AGE), enhanced activation of hexosamine, protein kinase C (PKC), and tissue renin-angiotensin system (RAS). The authors summarize that the overall effects of the metabolic abnormalities result in augmentation of ROS (reactive oxygen species) and RNS (reactive nitrogen species) production and associated oxidative and nitrosative damage, thereby leading to retinal vascular dysfunctions in DR. They also discuss the importance of metabolic memory caused by epigenetic changes such as a modified DNA methylation pattern, altered histone modifications of key regulatory proteins and altered microRNA (miRNA) expression leading to altered mitochondrial enzymes, damage to mitochondrial DNA, and altered mitochondrial ETC complexes. These result in superoxide formation, and depletion of antioxidants, eventually causing inflammation and apoptosis of retinal and endothelial cells during DR.

Oxidative stress associated with hyperlipidemia, hypertension, obesity, and IR, collectively referred to as Metabolic Syndrome (MS), is considered a risk factor for cardiovascular disease (CVD). Commonly used in traditional Asian and African medicine against hypertension, obesity, and hypercholesterolemia, in their work, using an MS rat model, I. Pérez-Torres et al. studied the antioxidant properties of *a medicinal herb*, *Hibiscus sabdariffa Linnaeus* (*HSL*), *in myocardial protection* against ischemia/reperfusion damage. The authors observed that cardiac mechanical performance, coronary vascular resistance, and activities of antioxidant enzymes were restored and oxidative damage was limited in HSL-treated rats compared to the untreated ones with MS. I. Pérez-Torres et al. concluded that HSL mediated myocardial protection during ischemia and reperfusion occurs through the antioxidant substances that it possesses such as PCA, anthocyanins, cyanidin-3-glucoside, quercetin, and polyphenols.

The study by M. Nitiéma et al. investigated whether oral administration of the ethyl acetate fraction of *Lannea microcarpa trunk barks* (*LMAE*) *corrects vascular dysfunction and angiotensin* (*Ang*) *II-induced hypertension in mice*. LMAE contains sterols, triterpenes, coumarins, and anthraquinone. Hemodynamic and echocardiographic parameters in vivo and vascular reactivity to acetylcholine (ACh) and CaCl_2_ ex vivo were studied on isolated aortas. Results showed that LMAE prevents Ang II-induced hypertension and vascular dysfunction through a reduction of oxidative stress linked to COX-2 and NOX-2 pathway and inhibition of calcium entry.

Cells shed small vesicles called microparticles (MPs) including exosomes, which are trapped in tissues or released into bodily fluids. They harbor proteins and surface antigens specific to cells they originate from. MPs also mediate critical actions in intercellular communication and transmit biological messages by acting as paracrine vehicles.

Of interest and because of their easy detection using a variety of techniques, circulating MPs were recognized as biomarkers for cell activation and cross talk between different cell types. High plasma numbers of MPs were reported in many cardiovascular and metabolic disturbances, which are closely associated with insulin resistance and low-grade inflammation. They have been associated with adverse effect on the heart and vasculature. The review by T. Benameur highlights the *involvement of microparticles in cardiovascular complications associated with diabetes* and discusses the molecular mechanisms that underpin the pathophysiological role of MPs in the onset and progression of cellular injury.

## References

[1] Sen S., Strappe P. M., O'Brien T. (2005). Gene transfer in endothelial dysfunction and hypertension. *Methods in Molecular Medicine*.

[2] Roy S., Khanna S., Nallu K., Hunt T. K., Sen C. K. (2006). Dermal wound healing is subject to redox control. *Molecular Therapy*.

[3] Galley J. C., Straub A. C. (2017). Redox control of vascular function. *Arteriosclerosis, Thrombosis, and Vascular Biology*.

[4] Rask-Madsen C., King G. L. (2007). Mechanisms of disease: endothelial dysfunction in insulin resistance and diabetes. *Nature Clinical Practice Endocrinology & Metabolism*.

